# Early COVID-19 pandemic’s toll on tuberculosis services, WHO European Region, January to June 2020

**DOI:** 10.2807/1560-7917.ES.2021.26.24.2100231

**Published:** 2021-06-17

**Authors:** Masoud Dara, Giorgi Kuchukhidze, Askar Yedilbayev, Ihor Perehinets, Tanja Schmidt, W. Leif Van Grinsven, Martin J. Boeree

**Affiliations:** 1 WHO Regional Office for Europe, Copenhagen, Denmark; 2 Department of Lung Diseases, Radboud Institute for Health Sciences, Radboud University Medical Center, Nijmegen, the Netherlands

**Keywords:** COVID-19, SARS-CoV-2, pandemic, tuberculosis, drug-resistant tuberculosis, public health and social measures, diagnosis, treatment

## Abstract

**Background:**

Essential health services, including for tuberculosis (TB), are being affected by public health and social measures (PHSM) introduced to control COVID-19. In many settings, TB resources, facilities and equipment are being redirected towards COVID-19 response.

**Aim:**

We sought to assess the COVID-19 pandemic’s impact on TB services in the World Health Organization (WHO) European Region.

**Methods:**

The fifty-three European Region Member States were asked to report qualitative and quantitative data in quarter one and two (Q1 and Q2) 2020. TB notifications were triangulated with the severity score on domestic movement restrictions to assess how they may have influenced TB detection.

**Results:**

Twenty-nine countries reported monthly TB notifications for the first half of 2019 and 2020. TB notifications decreased by 35.5% during Q2 2020 compared with Q2 2019, which is six-fold more than the average annual decrease of 5.1% documented during 2015–2019. The number of patients enrolled in rifampicin-resistant/multidrug-resistant TB treatment also decreased dramatically in Q2 2020, by 33.5%. The highest movement restriction severity score was observed between April and May 2020, which coincided with the highest observed decrease in TB notifications.

**Conclusion:**

A decrease in TB detection and enrolment to treatment may cause increases in TB burden and threatens the Region’s ability to reach the TB targets of the 2030 Sustainable Development Goals, still this might be mitigated with rapid restoration of TB services and the implementation of targeted interventions during periods with severe PHSM in place, such as those introduced in response to the COVID-19 pandemic.

## Introduction

On 30 January 2020, the World Health Organization (WHO) declared coronavirus disease (COVID-19) a Public Health Emergency of International Concern [1]. By mid-March 2020, the WHO European Region had become the epicentre of the pandemic, reporting over 40% of global confirmed cases. By the end of April 2020, more than 60% of global mortality from infection with the severe acute respiratory syndrome coronavirus 2 (SARS-CoV-2) occurred in Europe [2]. Before the COVID-19 pandemic, the WHO European Region was on track to achieve the 2020 targets for decreasing TB incidence and mortality despite having the highest rates of drug-resistant tuberculosis (TB) [3].

As the world focuses on tackling the COVID-19 pandemic, there is a risk that other essential health services and operations for long-standing health problems—including TB—will be hampered. While COVID-19 is a new disease, the world has been fighting TB epidemics for centuries. There are many lessons learnt from the TB response that can be applied to COVID-19 response. Many practices in TB response, such as triaging in the health centre setting, cough etiquette, contact tracing in the community and infection control in health centres and the community, including isolation, also benefit the COVID-19 response [4].

In 2020, countries in the WHO European Region experienced impact of COVID-19 on their health systems. As at September 2020, countries across the Region were in different stages of SARS-CoV-2 transmission, with many seeing a resurgence in the number of COVID-19 cases after successfully slowing outbreaks earlier in the year by applying a package of response measures. The pandemic caused many countries to respond by introducing public health and social measures (PHSM), including physical distancing, national and international movement restrictions, school and business closures as well as quarantine requirements, especially during the initial phase of local epidemics [5]. The PHSM in the Region were further intensified in the autumn and winter of 2020.

Planning and implementation of some measures, like local movement restrictions, can create an environment where in-person health service delivery, including diagnosis and treatment, are affected by the inability to reach healthcare facilities and service providers [6]. Furthermore, health services—including those offered by national TB programmes—are often actively engaged in the response to COVID-19. In many settings, TB and other health programme resources, facilities and equipment may have been directed towards the COVID-19 response [6,7]. These changes may include, but are not limited to, repurposing of healthcare staff, restructuring inpatient (and outpatient) services and using TB diagnostics services for COVID-19, e.g. the GeneXpert machines (Cepheid, Sunnyvale, California, United States (US)) that are used for initial diagnostic testing for TB and rifampicin resistance (as recommended by WHO) in many countries, especially in the eastern part of the Region. The pandemic may also bring other challenges to TB programmes, such as lack of funding for TB interventions, interruption in availability and access to medicines and supplies, reprioritisation of research and development, and administration of Bacillus Calmette–Guérin vaccination.

In order to identify the impact of the COVID-19 pandemic on TB services, the WHO headquarters initiated a concise, qualitative and quantitative survey to collect retrospective and prospective data from its Member States.

## Methods

### Tuberculosis data collection

As part of the annual, global TB data collection, additional qualitative data were collected from the WHO Member States through a survey conducted from April to June 2020, to enable WHO to report on how the COVID-19 pandemic has affected TB services. The survey included three main questions and additional 16 sub-questions related to changes in TB service delivery, self-isolation of TB patients and re-allocation of resources from TB services to COVID-19 testing and treatment (Supplementary Table S1).

As not all countries in the Region faced the COVID-19 pandemic at the same time and as they implemented various levels of restrictive measures and varying degrees of repurposing of services, the WHO Regional Office for Europe ran a more granular data enquiry from July to August 2020, asking countries to report monthly quantitative data on TB notifications from the first and second quarters (Q1 and Q2) of 2020, as well as monthly data from the same quarters of 2019 for comparative analysis. The WHO Regional Office for Europe sent a data call to 48 of its 53 member countries; no or very few TB cases are reported annually from Andorra, Luxembourg, Malta, Monaco and San Marino, and so the data call was not sent to these countries.

The data collection included the following indicators: (i) number of notified cases of all forms of TB (bacteriologically confirmed plus clinically diagnosed), (ii) new and relapse cases; (iii) number of patients that were put on TB treatment (all TB cases). In addition, 18 high-priority countries (HPC) for TB (Azerbaijan, Belarus, Bulgaria, Estonia, Georgia, Kazakhstan, Kyrgyzstan, Latvia, Lithuania, Moldova, Romania, Russia, Tajikistan, Turkey, Turkmenistan, Ukraine and Uzbekistan) were asked to report the number of cases with rifampicin-resistant TB and/or multidrug-resistant TB (RR-TB/MDR-TB) that began second-line treatment; and to report the TB treatment adherence and retention in care indicator (Supplementary Table S2).

### Data on public health and social measures

Data on the implementation of PHSM in response to the COVID-19 pandemic has been systematically captured by the emergency public health measures pillar (EPHM) of the COVID-19 Incident Management Support Team (IMST) at the WHO Regional Office for Europe. Data collection began in February 2020, and over 15,000 measures across 53 countries were recorded in the WHO/EURO PHSM database and classified following the PHSM taxonomy and glossary, as at 1 February 2021. PHSM data are collected from publicly available sources such as government and media articles; WHO country offices and emergency hub communications, where available, provide further information. The severity of countries’ PHSM is calculated following the PHSM Severity Index methodology. Data on six types of PHSM—mask wearing, school closures, business closures, gathering restrictions, domestic movement restrictions and limitations on international travel—are coded by country based on the severity and scope of the measures and are scored on a scale between zero and 100. In order to document the extent that COVID-19 notifications and PHSM may have influenced TB detection, country data on domestic movement restrictions collected by the Health Emergencies Programme of the WHO were triangulated with the WHO TB survey results. In order to quantify the data collected, each measure was scored based on an ordinal scale corresponding with the response policy’s degree of severity (Supplementary Table S3). Further information on the PHSM Severity Index data monitoring and collection, coding, calculation and applications is available in the methodology paper [8].

### Statistical analysis

Data analysis was performed using SAS v9.4 (Statistical Analysis System Institute Inc, Cary, NC, US). Descriptive statistics were used to calculate the proportions of practice of changing TB service delivery, introduction of self-isolation for TB patients and reallocation of resources from TB services to COVID-19 testing and treatment.

We compared monthly TB notification data from 2020 to monthly data from 2019 and evaluated the Regional, sub-Regional (European Union/European Economic Area (EU/EEA) vs non-EU/EEA) and country-level declines in TB notifications, enrolment to treatment and retention in care.

We linked TB notifications epidemic curve using the PHSM Severity Index scale for the category of domestic movement restriction between January and June 2020 to look at the relationship between these two.

### Ethical statement

This study was based on aggregated surveillance data submitted by the WHO Member States and ethical approval was not required.

## Results

### WHO survey on the impact of COVID-19 on tuberculosis service delivery

Of the 48 countries in the Region that were contacted, 44 responded to the WHO survey in the global TB database, assessing TB services during the COVID-19 pandemic (Table).

**Table:** Results of the World Health Organization survey on how the COVID-19 pandemic affected tuberculosis services in the WHO European Region countries, April–June 2020 (n = 44)

Survey questions	Total number (N)	Yes (n)	No (n)	Don’t know/did not respond (n)
1. Have any changes been made to how TB treatment services are delivered due to the COVID-19 pandemic?	44	25	18	1
1.1. Reduced frequency of outpatient visits to a health facility for treatment monitoring or collection of drugs for patients with drug-susceptible TB	44	22	20	2
1.2. Reduced frequency of outpatient visits to a health facility for treatment monitoring or collection of drugs for patients with multidrug or rifampicin-resistant TB	44	18	24	2
1.3. Amount of anti-TB drugs given to patients to take home increased to one month or more	44	18	22	4
1.4. TB patient can nominate another household member to collect anti-TB drugs from a health facility on their behalf	44	12	28	4
1.5. Home delivery of anti-TB drugs to TB patients	44	17	22	5
1.6. Expanded use of remote advice and support for TB patients	44	19	21	4
1.7. Reduced number of health facilities where outpatient TB treatment is provided for patients with drug-susceptible TB	44	10	33	1
1.8 Reduced number of health facilities where outpatient TB treatment is provided for patients with multidrug or rifampicin-resistant TB	44	8	35	1
1.9 Reduced number of hospitals where inpatient treatment is provided for patients with drug-susceptible TB	44	9	32	3
1.10. Reduced number of hospitals where inpatient treatment is provided for patients with multidrug or rifampicin-resistant TB	44	7	35	2
2. Have TB patients been asked to self-isolate at home?	44	20	21	3
3. Has there been any reallocation of resources from TB services to COVID-19 testing and treatment?	44	20	23	1
3.1. GeneXpert machines used for TB diagnosis reassigned for COVID-19 testing	44	6	36	2
3.2. Staff in the central unit of the NTP reassigned to other duties	44	14	29	1
3.3. NTP staff at subnational levels systematically reassigned to other duties	44	16	25	3
3.4. Budgets originally allocated for TB reallocated to the COVID-19 response	44	10	28	6

CI: confidence interval; COVID-19: coronavirus disease; NTP: National Tuberculosis Programme; TB: tuberculosis.

According to the survey, 25 of 44 countries had introduced changes to TB service delivery, with 10 countries and nine countries reducing the number of in-patient and outpatient TB facilities, respectively.

In 25 countries reporting changes in service delivery, common actions to mitigate the COVID-19 pandemic impact included reduced frequency of outpatient visits for treatment monitoring or collection of drugs, allowing TB patients to take a 1-month or more supply of anti-TB drugs home, expanded use of remote advice and support, and home delivery of anti-TB drugs.

Almost half of the reporting countries (20/44) had to reallocate TB resources to COVID-19 response, with six countries reporting the use of GeneXpert machines for COVID-19 testing instead of diagnostic testing for TB and 14 countries reporting National Tuberculosis Programme staff being reassigned for COVID-19–related duties.

### Indicators collected by the WHO Regional Office for Europe

**Figure 1:**
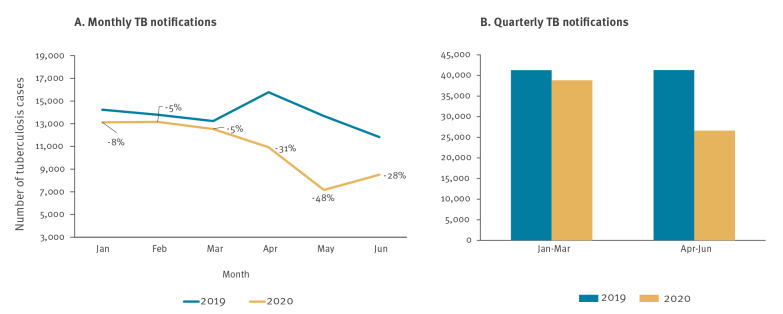
Comparison of monthly and quarterly notifications of new and relapse tuberculosis cases, World Health Organization European Region countries, January–June 2019 and 2020^a^

^a^ Data reported from 29 of 53 World Health Organization European Region countries.

Twenty-nine of 48 countries in the WHO European Region provided monthly data for the requested period and were included in the analysis. When comparing the monthly data reported in 2020 vs 2019, a substantial decrease in new and relapse TB case notifications was observed from April 2020 (−31%), with the most substantial decline in May (−48%) (Figure 1).

Overall, the number of detected TB cases fell by 5.6% in Q1 2020, and by 35.5% in Q2 2020, compared with the same periods in 2019. This decline was six-fold higher than the average trend of annual decrease observed during the last 5 years (Figure 1).

Monthly enrolment to anti-TB treatment showed a similar trend, with a considerable decrease observed from April 2020 (−32%), and with the most substantial decline in May (−48%).

Of those diagnosed with TB, 95–99% were enrolled to treatment, without significant variations in treatment coverage from January to June 2020.

**Figure 2:**
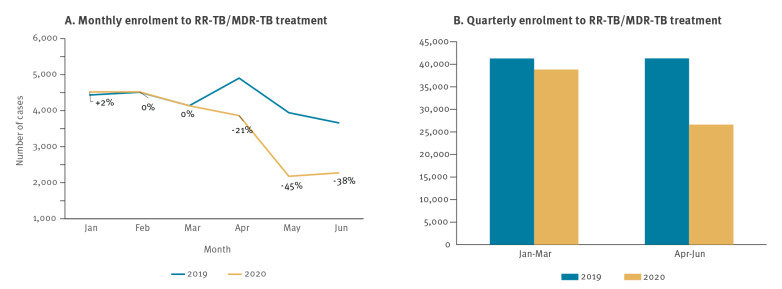
Comparison of (A) monthly and (B) quarterly enrolment to RR-TB/MDR-TB treatment, World Health Organization European Region high priority TB countries, January–June 2019 and 2020^a^

Fourteen of 18 HPC also reported monthly enrolment of RR-TB/MDR-TB cases to second-line anti-TB treatment. When comparing monthly data from 2020 vs 2019, we observed a similar trend from January to June 2020, with the same level of patients starting second-line TB treatment in January, followed by a sharp decline from March 2020 (−21%) and the most substantial decline in May (-45%) (Figure 2).

RR-TB/MDR-TB: rifampicin-resistant tuberculosis and/or multidrug-resistant tuberculosis, TB: tuberculosis.

^a^ Data reported from 14 of 18 World Health Organization European Region high priority TB countries.

Overall, the number of people enrolled to RR-TB/MDR-TB treatment increased by 0.7% Q1 2020 and decreased by 33.5% in Q2 2020, compared with the same periods in 2019 (Figure 2).

### Linking PHSM data to tuberculosis notifications

All WHO European Region countries implemented domestic movement restrictions to varying degrees of severity between January and June 2020, either at national or subnational levels. These often included limits on municipal travel, strict stay-at-home orders, curfews and reductions in public transport capacity. Strict domestic movement restrictions were implemented in many countries throughout the Region in March, which were lifted to varying degrees in the subsequent months. After the summer, many countries again increasingly recommended limiting people’s movement within the country.

**Figure 3:**
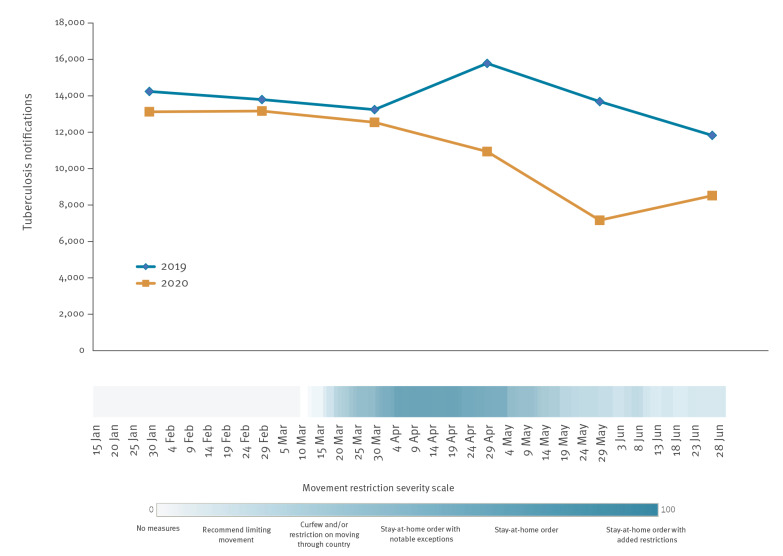
Monthly tuberculosis notifications by movement restrictions in 2020, World Health Organization European Region countries, January–June 2019 and 2020^a^

^a^ Data reported from 29 of 53 World Health Organization European Region countries.

After linking PHSM data to the TB notifications epidemic curve, we looked at the potential effect the restrictions on domestic movement had on TB notifications. The highest movement restriction severity score for 29 WHO European Region countries that reported monthly TB notifications was observed for the period April to May 2020, which coincided with the most substantial decrease in TB notifications (Figure 3).

**Figure 4:**
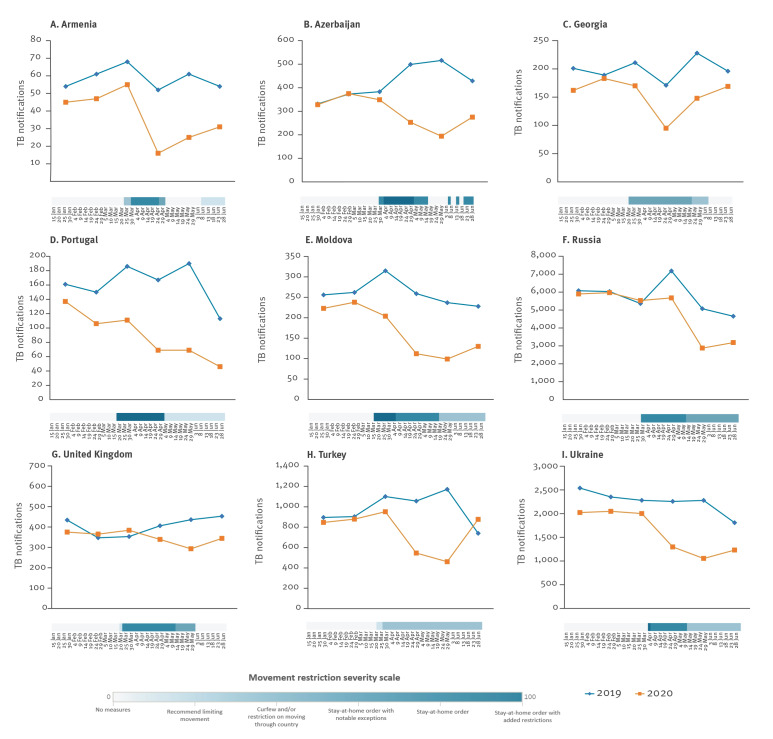
Monthly tuberculosis notifications over movement restrictions, selected WHO European Region countries January–June 2019 and 2020 (n = 9)^a^

^a^ Armenia, Azerbaijan, Georgia, Portugal, Moldova, Russia, Turkey, Ukraine and the United Kingdom.

The relationship between TB notifications and movement restrictions was more noticeable in a number of countries, namely in Armenia, Azerbaijan, Georgia, Portugal, Moldova, Russia, Turkey, Ukraine and the United Kingdom (Figure 4).

**Figure 5:**
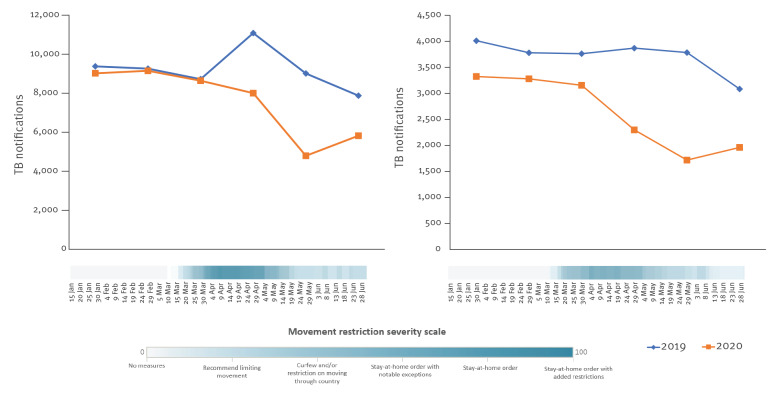
Monthly tuberculosis (TB) notifications by movement restrictions in 2020 in the countries reporting ‘some changes to TB services’ vs ‘no changes to TB services’, WHO European Region countries January–June 2019 and 2020^a^

When triangulating the PHSM data with the WHO survey results, we found no difference in the group of countries who reported ‘some changes to TB services’ vs a group of countries reporting ‘no changes to TB services’ (Figure 5).

TB: tuberculosis.

^a^ Data reported from 29 of 53 World Health Organization European Region countries.

## Discussion

During the period of 2015 to 2019, notification of new and relapse TB cases in the WHO European Region declined by 5.1% per year on average. This trend was in line with the annual decrease in the WHO estimated TB incidence for the Region (5.1%), resulting in a 19% reduction of the TB incidence rate between 2015 and 2019, which was the highest decline observed among all WHO Regions and on target for the 2020 End TB milestone [3]. The results of our survey show that there was a substantial decrease in monthly case detection and enrolment to treatment from April 2020. While the decrease of 5.6% in Q1 2020 was in line with the previous trend, an average 35.5% decrease in TB notification was observed in Q2 2020, compared with the same period in 2019.

Between 2015 and 2019, on average, 45,000 RR-TB/MDR-TB patients were enrolled to treatment annually in the WHO European Region, without showing a trend of decrease [9]. Since the start of the COVID-19 pandemic, enrolment to RR-TB/MDR-TB treatment decreased by 33.5% in Q2 2020, compared with the same period in 2019. Comparison of trends in TB notifications with the COVID-19–related movement restrictions implemented in 2020 show an association between the level of stringency of the domestic movement restrictions implemented and the decrease in TB notifications reported in most countries (29/53) that responded to the survey. While PHSMs are implemented to reduce COVID-19 transmission and flatten the epidemiological curve [9,10], our study points to an urgent need to adapt health services to ensure essential services are not hampered while the restrictive measures are in place. Decrease in enrolment of RR-TB/MDR-TB patients is equally concerning. Here other factors—including the repurposing of diagnostic and clinical services and the health workforce to focus on COVID-19, as well as changes in patients’ health-seeking behaviour—may play a role and require further study.

There is a need to introduce and/or scale up innovative and adaptive models of care to address the challenges posed by the COVID-19 pandemic and ensure quality and timely TB services are provided, despite the ongoing pandemic. Countries already reported some good examples, such as allowing TB patients to take a 1-month or more supply of anti-TB drugs home, expanded use of remote advice and support, and home delivery of anti-TB drugs.

Recent modelling works assessing the potential impact of COVID-19 movement restrictions illustrated that even short, 2–3-month COVID-19–related decreases in TB notifications can lead to long-lasting setbacks in TB response [11-14]. Different modelling scenarios showed that over the next 5 years, TB deaths in the WHO European Region could increase by 4–20%—which is up to 4,000 additional TB deaths—while TB incidence could see an increase of 3–9%.

Another mathematical model by Cilloni et al. on TB transmission in three high-burden countries, including one in the WHO European Region, concluded that a 3-month suspension of TB services, followed by 10 months to restore services to normal, would cause—over the next 5 years—an additional 4,350 TB cases and 1,340 TB deaths in Ukraine, 1.19 million TB cases and 361,000 TB deaths in India and 24,700 TB cases and 12,500 TB deaths in Kenya [13]. Therefore, our study findings call for consolidated interventions to mitigate the impact of the pandemic on TB prevention and care efforts in Europe and globally to reach the SDGs.

The Box presents a set of targeted interventions to address the challenges resulting from the public health and social measures that have been put in place to address the COVID-19 pandemic.

Box **Targeted interventions to address the negative impact of COVID-19 pandemic on tuberculosis services**

- Scaling up public health awareness campaigns so that individuals with TB symptoms would refer to health services.
- Continuing surveillance and monitoring of the TB epidemic, including the timely detection and management of TB outbreaks.
- Allocating resources for intensified contact tracing and testing for TB among risk groups and vulnerable populations.
- Improving infection prevention and control in healthcare facilities to decrease the risk of nosocomial COVID-19 transmission.
- Adopting and implementing the latest WHO guidance on treatment of TB and DR-TB, especially with the introduction of fully oral treatment regimens.
- Introducing and scaling up the use of digital health technologies to improve efficiencies, e.g. for booking appointments at healthcare facilities.
- Developing and implementing dual testing for SARS-CoV-2 and TB among symptomatic individuals and close contacts of TB patients.
- Scaling up people-centred models of care, e.g. outpatient/mobile clinic/home-based and community-based care.
- Introducing and/or scaling up video-supported treatment to enhance treatment retention [15].
- Prioritising and implementing tapered services for high-risk groups, particularly prisoners, migrants and people living with HIV, diabetes and other health conditions.

COVID-19: coronavirus disease; DR-TB: drug resistant TB; SARS-CoV-2: severe acute respiratory syndrome coronavirus 2; TB: tuberculosis; WHO: World Health Organization.

Apart from the importance of our findings, our study has several limitations. Monthly data were not available for all countries. This makes our assessment less sensitive to the introduction of COVID-19 control measures in each country. Further, limited data granularity does not allow identifying TB services for particular risk groups, and vulnerable populations may have been more seriously affected by the COVID-19 pandemic.

The PHSM Severity Index captures policies implemented by countries for a selected number of PHSM. Additionally, the domestic movement data capture government recommendations and requirements, not the enforcement or compliance of measures, nor the movement patterns of a population. Given the fact that PHSM are commonly implemented in packages, it is difficult to disentangle the effects of one type of measure in particular. While every effort has been made to ensure the accuracy and completeness of the PHSM Severity Index through a rigorous systematic approach to data collection, categorisation, coding and analysis—as well as through various validation exercises—the ongoing COVID-19 pandemic is a fast-paced, dynamic situation and the possibility for errors or omissions in data can never be completely eliminated.

## Conclusions

In conclusion, we found a substantial decrease in TB notifications in Q2 2020 in the WHO European Region. This delay or lack of diagnosis can lead to ongoing transmission of the disease to close contacts, increased severity of TB disease and a potential increase in case fatality. Urgent action and innovative solutions need to be implemented to catch up with the delays in diagnosis, treatment enrolment and retention during the pandemic. More research is needed to assess the effects of the COVID-19 pandemic and resulting PHSM on specific diseases, including TB, HIV, malaria and other communicable and non-communicable diseases.

National health programmes and health authorities need to work with community representatives and partners across all sectors to ensure that efforts to diagnose, treat and care for TB patients are not undermined during the pandemic. Revamping political commitment, allocating adequate national and international resources, and implementing innovative approaches are crucial to avoid losing the achievements of the past decade, so that the world is closer to ending the COVID-19 pandemic as well as the TB epidemic, as soon as possible.

## Supplementary Data

72000 Supplement
