# Erratum for Euro Surveill. 2021;26(24)

**DOI:** 10.2807/1560-7917.ES.2021.26.26.210701e

**Published:** 2021-07-01

**Authors:** Eurosurveillance editorial team

**Affiliations:** 1 European Centre for Disease Prevention and Control (ECDC), Stockholm, Sweden

In the article ‘Early COVID-19 pandemic’s toll on tuberculosis services, WHO European Region, January to June 2020’ by Dara et al. published on 17 June 2021, Panel B in Figure 2 was wrong. This was replaced with the correct one on 1 July 2021. We apologise for any inconvenience this error may have caused.

