# Supplementary material for: Early COVID-19 pandemic’s toll on tuberculosis services, WHO European Region, January to June 2020
Source: Euro Surveill. 2021 Jun 17;26(24):2100231. doi: 10.2807/1560-7917.ES.2021.26.24.2100231 (PMC8212590; doi:10.2807/1560-7917.ES.2021.26.24.2100231)
Supplement: Supplement [file 21-00231_DARA_supplement.pdf]

Supplement 1

Restrictions on domestic movement	0 – No measures<br>1 – Recommend not leaving house and/or recommend limiting domestic movement<br>2 – Restriction on domestic movement (such as a ban on travelling between or into certain regions, outside a certain radius from place of residence, or night-time curfew)<br>3 – Requirement not to leave house with exceptions for the following: essential activities (grocery shopping and essential trips), daily exercise, limited social interactions (visiting family or friends) or travel to other places of residence<br>4 – Requirement not to leave house with exception only for essential activities (grocery shopping, pharmacy or essential trips)<br>5 – Requirement not to leave house with exceptions for essential activities (grocery shopping or essential trips) allowed only under certain conditions (such as being allowed to leave house only once a week, during designated timeslot or only one household member can leave at a time)	0 – Targeted<br>(subnational or groups of people)<br>1 – General (national)
